# RETRACTED: Efficacy and risk of sexual orientation change efforts: retrospective analysis of 125 exposed men

**DOI:** 10.12688/f1000research.161433.1

**Published:** 2025-02-04

**Authors:** F1000 Editorial Office

**Affiliations:** 1 F1000, London, UK

**Keywords:** sexual orientation change, psychosocial health, marriage, ex-gay

## Abstract

Retraction Notice (4th February 2025): The article by Sullins DP, Rosik CH and Santero P: ‘Efficacy and risk of sexual orientation change efforts: a retrospective analysis of 125 exposed men' (https://doi.org/10.12688/f1000research.51209.2) has been retracted from F1000Research by the F1000 Editorial Team.

A previous version of this article published on F1000Research has also been retracted (https://doi.org/10.12688/f1000research.51209.1).

After publication, concerns were raised to the F1000 Editorial Team regarding the data underlying this research. In accordance with F1000 policies, the F1000 Editorial Team investigated these concerns and conducted two independent external reviews. The reviews concluded that the methodology, and subsequent statistical analysis, employed by the study cannot support the conclusions around efficacy and effectiveness. Considering this, the F1000 Editorial Team has been unable to confirm the integrity of the study design and the article’s conclusions.

Therefore, the F1000 Editorial Team no longer has confidence in the reported conclusions and has decided to retract the article.

The authors listed in the publication have been informed. The authors do not agree with the retraction.

We have been informed in our decision-making by our editorial policies and the COPE guidelines.

